# HPV16 variant lineage, clinical stage, and survival in women with invasive cervical cancer

**DOI:** 10.1186/1750-9378-6-19

**Published:** 2011-10-28

**Authors:** Rosemary E Zuna, Erin Tuller, Nicolas Wentzensen, Cara Mathews, Richard A Allen, Rebecca Shanesmith, S Terence Dunn, Michael A Gold, Sophia S Wang, Joan Walker, Mark Schiffman

**Affiliations:** 1 Department of Pathology, University of Oklahoma Health Sciences Center, 940 SL Young Blvd, Oklahoma City, Oklahoma, 73104-5042, USA; 2 Department of Obstetrics and Gynecology, Division of Gynecologic Oncology, University of Oklahoma Health Sciences Center, 800 NE 10th St. Suite 2001-2, Oklahoma City, Oklahoma, 73104-5418, USA; 3 Division of Cancer Epidemiology and Genetics, National Cancer Institute, 6120 Executive Blvd, Room 5024, Rockville, Maryland 20852-7234, USA; 4 Department of Obstetrics and Gynecology, University of Missouri Health System, 115 Business Loop 70W, Columbia, Missouri 56203-3244, USA; 5 Department of Pathology and Laboratory Medicine, Tulane University Health Sciences, 1430 Tulane Avenue SL-79, New Orleans, Louisiana 70112-2699, USA; 6 Obstetrics and Gynecology Department, Vanderbilt University Medical Center, 1161 21st Ave S, Nashville, Tennessee 37232-0011, USA; 7 Department of Cancer Etiology, City of Hope and the Beckman Institute, 1500 East Duarte Rd, Duarte, CA 91010-3012, USA

**Keywords:** cervical neoplasms, human papillomavirus 16, HPV16 variants

## Abstract

**Background:**

HPV16 variants are associated with different risks for development of CIN3 and invasive cancer, although all are carcinogenic. The relationship of HPV 16 variants to cancer survival has not been studied.

**Methods:**

155 HPV16-positive cervical cancers were categorized according to European and non-European variant patterns by DNA sequencing of the *E6 *open reading frame. Clinico-pathologic parameters and clinical outcome were collected by chart review and death registry data.

**Results:**

Of the 155 women (mean age 44.7 years; median follow-up 26.7 months), 85.2% harbored European variants while 14.8% had non-European sequences. HPV16 variants differed by histologic cell type (p = 0.03) and stage (1 vs. 2+; p = 0.03). Overall, 107 women (68.0%) were alive with no evidence of cancer, 42 (27.1%) died from cervical cancer, 2 (1.3%) were alive with cervical cancer, and 4 (2.6%) died of other causes. Death due to cervical cancer was associated with European variant status (p < 0.01). While 31% of women harboring tumors with European variants died from cervical cancer during follow-up, only 1 of 23 (4.4%) non-European cases died of cancer. The better survival for non-European cases was partly mediated by lower stage at diagnosis.

**Conclusions:**

Overall, invasive cervical cancers with non-European variants showed a less aggressive behavior than those with European variants. These findings should be replicated in a population with more non-European cases.

## Background

While the association of HPV genotypes and cervical carcinoma is well established, the reasons that only a subset of lesions associated with high-risk genotypes progress to invasive cancer remain elusive. Influential variables likely include individual host factors, viral differences or combinations of both.

One variable that could contribute to differences in biological behavior of HPV- associated lesions is that of HPV DNA sequence variation. HPV16, which is the most prevalent HPV genotype and is associated with approximately half of cervical cancers worldwide [1], has well-documented DNA sequence variants. HPV variants are defined as isolates with primary DNA sequence differences that total no more than 2% of the *L1 *open reading frame (ORF) of the prototype sequence [2]. The sequence variations of the HPV16 *E6 *ORF have been found to correctly classify the HPV16-variants [3]. Two major categories of HPV16 variants have been defined: European (E) and Non-European (NE) patterns that appear to have evolved principally on a geographic basis [4]. A number of studies have suggested that HPV16 variants differ in risk for progression to high grade intraepithelial lesions [5-7] and in their association with the development of cancer [8-12]. Overall, the 16-NE variants have shown an increased risk for progression to CIN2 or greater when compared with 16-E.

We have previously reported the patterns of HPV16 variants in the spectrum of cervical lesions in our population [11]. While our study confirmed the increased risk for progression to carcinoma associated with 16-NE variants, 16-E patterns were found in the majority of HPV16 lesions, including cancers. We attributed this to the dominance of the 16-E patterns in our population and to the fact that all major variant categories can be found in cancers.

In this follow-up study, we address the question of whether HPV16 variant status influences clinical patterns and/or prognosis in fully evolved cervical cancers. We performed a cross-sectional analysis of the clinico-pathologic characteristics of an unselected, prospectively accumulated population of women with HPV16-positive invasive cervical cancer from the central United States. The tumors were categorized according to histologic cell type and HPV16 variant category. We also correlated the clinico-pathologic characteristics and clinical follow-up with HPV16-variant category in an effort to evaluate the possible association of the HPV16 variants with clinical behavior in cervical cancers.

A similar analysis was performed for the largest subpopulation, i.e., HPV16-positive squamous cell cancers harboring European variants, in an effort to identify differing patterns of biologic behavior within sub-lineages of this dominant category.

## Results

### Patient Demographics

The population of 155 women with HPV16- associated cancers had a mean age of 44.7 years ± 1.0 (SEM), ranging from 20-76 years. Median follow-up was 26.7 ± 24.5 months (mean = 31.5 ± 2.0 months). Overall, 107 women (68.0%) were alive with no evidence of cancer, 42 (27.1%) died from cervical cancer, 2 (1.3%) were alive with cervical cancer, and 4 (2.6%) had died of other causes. Of these cases, 132 (85.2%) harbored 16-E variants while 23 (14.8%) had 16-NE patterns (AA = 17 cancers, NA-1 = 3, AF1 and AF2 = 3). The patient characteristics for this population, sorted according to variant status, are summarized in Table 1.

**Table 1:** Clinicopathologic Features of 149 Cervical Cancer Cases

TRAIT	CATEGORY	EUROPEAN (%^1^) n = 127	NON EUROPEAN (%^1^) n = 22	TOTAL (%^2^)	P-VALUE^3^ n = 149
	Squamous Carcinoma	119 (87.5)	17 (12.5)	136 (91.3)	
CELL TYPE	Adenocarcinoma	5 (55.6)	4 (44.4)	9 (6.0)	0.03
	Adenosquamous Ca	3 (75.0)	1 (25.0)	4 (2.7)	

					

AGE GROUP	20-40	46 (78.0)	13 (22.0)	59 (39.6)	
(YEARS)	41-50	46 (92.0)	4 (8.0)	50 (33.6)	0.13
	51-72	35 (87.5)	5 (12.5)	40 (26.8)	

					

	White	102 (88.7)	13 (11.3)	115 (77.7)	
RACE	Black	4 (80.0)	1 (20.0)	5 (3.4)	0.11
	Hispanic	7 (63.6)	4 (36.4)	11 (7.4)	
	Native American	10 (71.4)	4 (28.6)	14 (9.5)	
	Asian	2 (100)	0	2 (1.4)	
	Other	1 (100)	0	1 (0.7)	
	Unknown	1 -	-	1 -	

					

	1A	14 (87.5)	2 (12.5)	16 (11.1)	
STAGE (FIGO)	1B1	42 (75.0)	14 (25.0)	56 (38.9)	0.16
	1B2	16 (88.9)	2 (11.1)	18 (12.5)	
	2	22 (91.7)	2 (8.3)	24 (16.7)	
	3	18 (94.7)	1 (5.3)	19 (13.2)	
	4	11 (8.9)	0	11 (7.6)	
	Recurrent/Unstaged	4 -	1 -	5 -	

					

	0	6 (85.7)	1 (14.3)	7 (4.9)	
GRAVIDITY	1-3	73 (82.0)	16 (18.0)	89 (61.8)	0.13
	>3	45 (93.8)	3 (6.3)	48 (33.3)	
	ND^4^	3 -	2 -	5 -	

					

PARAMETRIA^5^	Negative	53 (77.9)	15 (22.1)	68 (60.7)	0.12
	Positive	40 (90.9)	4 (9.1)	44 (39.3)	
	ND	34 -	3 -	37 -	

					

Table 1 (cont'd)					
	<1	16 (84.2)	3 (15.8)	19 (14.6)	
TUMOR SIZE^5 ^(cm)	1-4	42 (76.4)	13 (23.6)	55 (42.3)	0.1
	>4	51 (91.1)	5 (8.9)	56 (43.1)	
	ND	18 -	1 -	19 -	

					

	< 3	15 (93.8)	1 (6.3)	16 (19.3)	
INVASION DEPTH^6^	3.1 - 5.0	10 (83.3)	2 (16.7)	12 (14.5)	0.21
(mm)	>5	40 (72.7)	15 (27.3)	55 (66.3)	
	ND	62 -	4 -	66 -	

					

	Positive	40 (74.1)	14 (25.9)	54 (62.1)	
VSI^6^	Negative	29 (87.9)	4 (12.1)	33 (37.9)	0.17
	ND	58 -	4 -	62 -	

					

RESECTION	Positive	11 (91.7)	1 (8.3)	12 (14.6)	
MARGINS^6^	Negative	53 (75.7)	17 (24.3)	70 (85.4)	0.29
	ND	63 -	4 -	67 -	

					

LYMPH NODES^7^	Negative	48 (85.7)	8 (14.3)	56 (50.4)	
	Positive	44 (80.0)	11 (20.0)	55 (49.6)	0.46
	ND	35 -	3 -	38 -	

					

	NED	86 (80.4)	21 (19.6)	107 (71.8)	
STATUS^8^	DOD	41 (97.6)	1 (2.4)	42 (28.2)	0.008

^1^: Percentage calculated across.

^2^: Percentage calculated down.

^3^: Fisher's Exact Test

^4^: ND: No data available

^5^: Parametrial assessment and tumor size were determined by pathologic analysis of a radical hysterectomy or as part of a clinical staging procedure.

^6^: Depth of invasion, margin status and status of vascular space invasion were available only for women with early stage tumors who had a conization or hysterectomy.

^7^: Lymph node dissections were routinely performed as part of radical hysterectomy. It is not standard for higher stage women although cervical biopsy with lymph node dissection was performed for some higher stage women.

^8^: Status: NED = No evidence of cervical cancer at last follow-up; DOD = Dead of cervical cancer

### Clinico-pathologic variables

Of 149 women with definite clinical outcome, the overwhelming majority, 91.3%, were diagnosed with SCC. 16-E variants were identified in 119 (87.5%) SCC lesions. These accounted for 93.7% of all 16-E variant cases in this population. Although SCC cancers harboring 16-E variants dominated in this population, and 16-E cases were more frequent in all other histologic categories as well, the percentage of 16-NE variants ranged from 44.4% (4 of 9) of CAC compared with 25% (1 of 4) for ASC and 12.5% (17 of 136) for SCC. The distribution of individual 16-NE variant categories for histologic cell type is as follows: AA = 12 SCC and 4 CAC; NA-1 = 3 SCC; AF1 = 1 SCC and AF2 = 1 SCC and 1 ASC. Despite the disparity in case numbers, the differences in the distribution of HPV16-variants among the histologic cell types were statistically significant (p = 0.03).

There was no significant difference in the distribution of HPV16 variants according to tumor size, depth of invasion, vascular space invasion, lymph node involvement, parametrial involvement, or status of surgical margins. Similarly, none of the clinical parameters showed a significant difference associated with 16-variant status including race/ethnicity, age group, FIGO stage and number of pregnancies.

**Figure 1:**
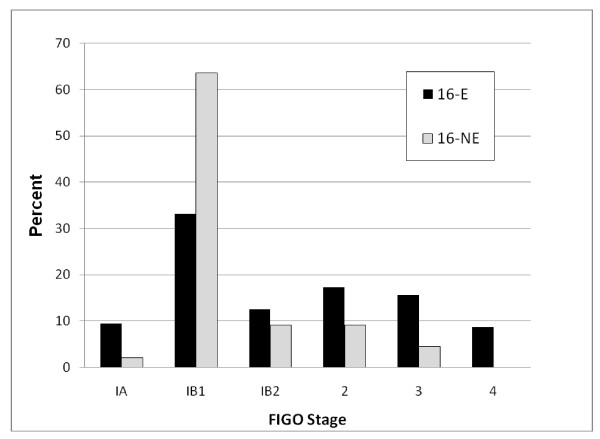
**Distribution of FIGO stage for women presenting with cervical carcinoma according to 16-variant status**.

Although significant differences were not identified between the 16-E and 16-NE variant groups for the above prognostic variables affecting survival in cervical cancer patients, there were interesting trends among 16-NE cases for some parameters that may reveal significance in a larger study with a larger population of 16-NE cases. These variables include younger age, early stage at presentation, three or fewer pregnancies, race/ethnicity other than white, depth of stromal invasion greater than 5 mm, and the presence of lymphovascular space invasion for 16-NE patients. While not statistically significant (p_trend _= 0.13), the modal peak for 16-NE cases was the 31-40 age group (mean = 41.4 ± 2.6 years; median 38 years) compared with the 41-50 group for the 16-E cases (mean = 45.3 ± 1.0; median 45 years). Figure 1 illustrates that the majority of women with 16-NE lesions presented at stage IB1 while those with 16-E lesions presented at a more variable stage (p_trend _= 0.02).

Because of the uneven distribution of cases in this study, a second analysis was performed that was restricted to the 123 cases of SCC associated with European variants. This population had a mean age of 45.9 years ± 1.1 (SEM), ranging from 20 to 76 years of age. Median follow-up was 28 months ± 22.4 (mean 33.0 ± 2.0). This population was divided into three categories as follows based on subgroups of European variants: European prototype (16-EP) (N = 53), European variants with a small number of nucleotide changes compared to prototype (16-EV) (N = 21), and those European variants harboring the 350 T>G nucleotide change (N = 49).

The 16-EV category has been previously described [11] and is similar to the European prototype with the addition of one or more single nucleotide changes. In this report, we subdivided the 16-EV category into two groups based on the presence or absence of the common 350T>G nucleotide change. In the first, termed 16-EV here, the nucleotide pattern differed from prototype by one nucleotide alteration. The location of the nucleotide change was variable and only the 178 T>G (sometimes termed the As variant) alteration (n = 4) was found in more than one case. The second pattern (16-EV-G) was defined by the presence of the European 350 T>G alteration as a single nucleotide change or with a small number of additional single nucleotide changes [11]. For example, three cases in this group showed a 131A>G change in addition to 350T>G. Because of the frequency of the 350T>G pattern, this group of 16-EV tumors was analysed separately in this study in an effort to clarify the clinical significance of this change. The 16-EV-G category specifically excludes the 16-NE variants that contain this same nucleotide 350 alteration. The clinico-pathologic characteristics of this population are shown in Table 2.

**Table 2:** Clinicopathologic Features of 119 HPV16-Positive Squamous Cell Carcinoma Cases with European Variants

TRAIT	CATEGORY	16-EP (%^1^) n = 52	16-EV (%^1^) n = 20	16-EV-G (%^1^) n = 47	Total (%^2^) n = 119	P-VALUE^3^
	Non-keratinizing	26 (41.9)	12 (19.4)	24 (38.7)	62 (57.4)	
CELL TYPE	Keratinizing	21 (45.7)	5 (10.9)	20 (43.5)	46 (42.6)	0.53
	Squamous, NOS^4^	5 -	3 -	3 -	11 -	

						

AGE GROUP	20-40	15 (37.5)	5 (12.5)	20 (50.0)	40 (33.6)	
	41-50	20 (45.5)	11 (25.0)	13 (29.5)	44 (37.0)	0.26
	51-72	17 (48.6)	4 (11.4)	14 (40.0)	35 (29.4)	

						

RACE	White	44 (45.8	15 (15.6)	35 (38.5)	94 (76.1)	
	Black	2 (50.0)	2 (50.0)	0	4 (3.4)	0.07
	Hispanic	2 (28.6)	0	5 (71.4)	7 (5.9)	
	Native American	3 (30.0)	1 (10.0)	6 (60.0)	10 (8.5)	
	Asian	1 (50.0)	1 (50.0)	0	2 (1.7)	
	Other	0 -	1 (100)	0	1 (0.8)	
	ND^5^	0 -	0 -	1 -	1 -	

						

	1A	6 (42.9)	2 (14.3)	6 (42.9)	14 (12.2)	
STAGE (FIGO)	1B1	16 (44.4)	8 (22.2)	12 (33.3)	36 (31.3)	0.75
	1B2	4 (28.6)	3 (21.4)	7 (50.0)	14 (12.2)	
	2	10 (45.5)	1 (4.5)	11 (50.0)	22 (19.1)	
	3	9 (50.0)	3 (16.7)	6 (33.3)	18 (15.6)	
	4	5 (45.5)	3 (27.3)	3 (27.3)	11 (9.6)	
	Recurrent/Unstaged	2 -	0 -	2 -	4 -	

						

	0	4 (80.0)	0 -	1 (20.0	5 (4.3)	
GRAVIDITY	1-3	24 (35.8)	11 (16.4)	32 (47.8)	67 (57.8)	0.18
	>3	22 (50.0)	9 (20.5)	13 (29.5)	44 (37.9)	
	ND^5^	2 -	0 -	1 -	3 -	

						

PARAMETRIA^6^	Negative	20 (43.5)	8 (17.4)	18 (39.1)	46 (53.5)	
	Positive	19 (47.5)	6 (15.0)	15 (37.5)	40 (46.6)	0.92
	ND	13 -	6 -	14 -	33 -	

						

Table 2 (cont'd)						
	<1	6 (40.0)	2 (13.3)	7 (46.7)	15 (14.7)	
TUMOR SIZE^6 ^(cm)	1-4	18 (46.2)	7 (17.9)	14 (35.9)	39 (38.2)	0.96
	>4	21 (43.8)	7 (14.6)	20 (41.7)	48 (47.1)	
	ND	7 -	4 -	6 -	17 -	

						

	< 3	3 (23.1)	2 (15.4)	8 (61.5)	13 (22.0)	
INVASION DEPTH ^7^	3.1 - 5.0	5 (62.5)	0	3 (37.5)	8 (13.3)	0.24
(mm)	>5	19 (50.0)	7 (18.4)	12 (31.6)	38 (64.4)	
	ND	25 -	11 -	24 -	60 -	

						

	Positive	15 (40.5)	6 (16.2)	16 (43.2)	37 (59.7)	
VSI^7^	Negative	12 (48.0)	3 (12.0)	10 (40.0)	25 (40.3)	0.83
	ND	25 -	11 -	21 -	57 -	

						

RESECTION	Positive	7 (63.6)	1 (9.1)	3 (27.3)	11 (19.3)	
MARGINS^7^	Negative	19 (41.3)	8 (17.4)	19 (41.3)	46 (80.7)	0.49
	ND	26 -	11 -	25 -	65 -	

						

	Negative	20 (47.6)	4 (9.5)	18 (42.9)	42 (49.4)	
LN STATUS^8^	Positive	18 (41.9)	8 (18.6)	17 (39.5)	43 (50.6)	0.55
	ND	14 -	8 -	12 -	34 -	

						

	NED	34 42.5)	13 (16.3)	33 (41.3)	80 (67.2)	
STATUS^9^	DOD	18 46.2)	7 (17.9)	14 (35.9)	39 (32.8)	0.87

^1^: Percentage calculated across.

^2^: Percentage calculated down.

^3^: Fisher's exact test

^4^: Squamous, NOS: Women with very small tumors or tiny biopsies were difficult categorize as to cell type and were not included in these analyses.

^5^: ND: No data available

^6^: Tumor size and parametrial assessment were determined by pathologic evaluation of a radical hysterectomy or as part of a clinical staging procedure.

^7^: Depth of invasion, margin status and status of vascular space invasion were available only for women with early stage tumors who had a conization or hysterectomy.

^8^: Lymph node dissections were routinely performed as part of radical hysterectomy. It is not standard for higher stage women although cervical biopsy with lymph node dissection was performed for some higher stage women.

^9^: Status: NED = No evidence of cervical cancer; DOD = Dead of cervical cancer

Cross-sectional analysis restricted to the subgroup of 123 women with SCC harboring European variants failed to demonstrate significant differences among the various clinical and pathologic traits for the different variant categories (Table 2). The 16-EP and 16-EV-G categories had similar characteristics. The 16-EV group was somewhat divergent but the numbers were small and the differences not significant. A comparison of 16-EV-G cases relative to all other 16-E SCC also showed no significant differences (data not shown).

### Clinical Follow-up

After a median follow-up of 29.1 months ± 23.3, death due to cervical cancer was highly associated with 16-E variant status (p < 0.01). While 31% (41/127) of women harboring tumors with 16-E variants died from cervical cancer, only 1 of 23 (4.4%) 16-NE cases died of cervical cancer. There were no differences in follow-up status or total follow-up time within the subgroups of 16-E squamous cancers.

We attempted to explain the survival differences by adjustment (by stratification or restriction) for possible factors such as stage at diagnosis or age, which showed a relationship both to variant status and outcome. Even within Stage 1 cancers, however, for which survival was favorable, tumors with 16-E variants tended to have somewhat worse outcomes than those with 16-NE variants (data not shown).

## Discussion

The rationale behind the possibility of biological differences associated with variant status is related to alterations in primary HPV DNA sequence that may alter control elements or affect the function of translated proteins that interact with the host. For example, the most common *E6 *sequence variation from prototype described in the 16-E-variants is 350 T> G that results in a L83V amino acid change in the E6 protein. Such amino acid changes can potentially alter tertiary structure and may influence protein function. *In vitro *studies of different HPV16-variants have suggested differences in biological activity [13], but an *in vivo *mechanism of enhanced biological activity has not been described. To date, evidence for different biological effects related to the sequence variants in patients has accumulated on an epidemiologic basis.

The increased risk for progression of 16-NE cervical lesions to high grade CIN and carcinoma has been attributed in part to reports that 16-NE variants are associated with a higher rate of HPV16 persistence and development of high-grade intraepithelial cervical lesions [6,7]. In this report, we explored the question of whether this increased risk for progression associated with 16-NE variants also correlates with increased aggressiveness of fully evolved invasive cancers. Our data for 155 HPV16-positive cervical cancer cases followed for a median of 29.1 months does not support this hypothesis. In fact, the women with tumors harboring 16-E variants had a statistically increased risk of death due to cervical cancer. A major problem in interpreting these results, however, is the skewed pattern of our cases, with strong predominance of squamous cancers and 16-E patterns. This imbalance could mask any possible difference in the behavior associated with the 16-NE lesions. For example, 16-NE variants, particularly the AA variant, have been reported to be associated with CAC [9,10] which have a more aggressive course than SCC in some reports [14,15]. In this population, however, only 14 (9.0%) HPV16-positive cases were CAC or ASC.

Survival in cervical cancer patients is related primarily to stage at diagnosis, tumor size, depth of cervical stromal invasion, parametrial involvement, lymphovascular space invasion, and lymph node status [16]. Histologic cell type [17] as well as HPV genotype, especially HPV18 [18] have also been reported as prognostic factors.

While survival in this study was correlated with 16-E variant status, there are clues to suggest that this may be an overly simplistic impression given the disproportionate number of cases in the two groups. In this study most of the 16-NE cancer patients presented at a young age and at an early stage (1B1). Additionally, these data showed non-significant trends in which 16-NE lesions had larger size at presentation with increased rate of vascular space invasion and lymph node metastases. It is therefore conceivable that the small number of 16-NE lesions in this population has resulted in a non-representative distribution of cases. Similarly, it is not clear whether this pattern is representative of all 16-NE cases or if our population is simply anomalous due to small numbers of 16-NE cases. Conceivably, our young 16-NE cases represent a detection bias in which younger women with cancer were identified because of screening while the older 16-E population presented with signs and symptoms of cancer. The relatively small proportion of 16-NE cases could thus result in anomalous results. On the other hand, these results raise the possibility that 16-NE variants have a preferential role in progression to malignancy but do not differentially influence clinical behavior in fully evolved cancers. When we analyzed a subpopulation of cancer cases representing the dominant group of HPV16 European sequence patterns, we were unable to show a difference in clinical behavior based on subcategories of 16-E variants.

## Conclusions

While our earlier study showed a significant association of 16-NE variant status with progression to high grade CIN and invasive cancer in cases with a wide spectrum of cervical disease[11], we have not been able demonstrate more aggressive behavior for actual invasive carcinomas associated with these variants. These results, however, should be interpreted with caution due to the limited number of 16-NE cases in this population. A larger study with a larger population of 16-NE cases is needed to clarify these issues.

## Methods

### Patient Population

From 1999-2009, 319 unselected cases of primary, metastatic and recurrent invasive cervical carcinoma were prospectively entered into this study based on the collection of a liquid-based cytologic sample that was tested for HPV DNA and a histologic diagnosis of invasive cervical cancer. The population seen at our institution is variably screened for cervical cancer so that some cancer patients were identified by routine screening while others presented with clinical signs and symptoms related to cervical cancer. Included in this total were 164 cases from the SUCCEED study [19] and 85 cases that were included in previous reports [20]. These women were staged according to FIGO (International Federation of Gynecology and Obstetrics, 2000) and treated at the OU Medical Center in Oklahoma City using standard therapies of surgery, radiation, and chemotherapy as indicated by individual patient characteristics. Of the 167 HPV16-positive cancer cases, 155 were available for variant analysis. Because 80 (51.6%) women did not undergo total hysterectomy and 40 (25.8%) did not have a lymphadenectomy, some pathologic variables were not evaluable in all cases. Follow-up was determined by chart review and death records for the state of Oklahoma. In addition to clinico-pathologic parameters, we compared over-all follow-up taking into account clinical stage at diagnosis, race, age, and censoring due to loss-to-follow-up. Statistical analyses reported below were performed using the population of women (n = 149) with defined outcome (i.e., no evidence of disease or dead of cervical cancer). This study was performed with the approval of the Institutional Review Board for the University of Oklahoma Health Sciences Center.

### Pathologic Categorization

All cancer diagnoses were confirmed histologically and the cell type was determined using routine hematoxylin and eosin staining supplemented by any special stains performed at the time of initial diagnosis. The tumor cell types were categorized using standard histologic criteria [21]. Squamous cell carcinomas (SCC) were designated as "large cell keratinizing" when at least one well-formed keratin pearl was identified [22] and "large cell non-keratinizing" when no pearls were identified. Squamous tumors that were very small or for which only small biopsies were available, were listed as "SCC, not otherwise specified".

The major histologic categories included SCC (n = 141), both keratinizing and non-keratinizing patterns, adenocarcinoma (CAC) (n = 10), and adenosquamous carcinoma (ASC) (n = 4). There were no HPV16-positive small cell neuroendocrine carcinomas in this population.

### HPV Testing Using L1 Consensus Primers

Liquid-based cytologic samples were collected in PreservCyt^® ^(Hologic, Malborough, MA) at the time of clinical evaluation and the cellular DNA was extracted using the QIAamp^® ^DNA Blood and Tissue Mini Kit (Qiagen, Valencia, CA). HPV genotyping was performed using the reverse line blot/Linear Array® HPV Genotyping Test (Roche Molecular Systems, Alameda, CA), as previously described [20].

### Variant Analysis

Aliquots of cellular DNA from HPV16-positive tumor samples were analyzed for HPV16 *E6 *variant category using PCR amplification and bidirectional PCR-based fluorescent dideoxy chain termination sequencing using the same primers used for initial amplification, as previously described [11].

Sequence alignments were performed using CLUSTALW [23]. Only nucleotide changes verified as occurring on both strands were accepted.

An HPV16-variant category was assigned for each case using the prototype nucleotide sequence for HPV16 [24] (modified as HPV16R [25]) as well as published HPV16 *E6 *sequence patterns that define the different variants [3]. The 16-E category included the prototype sequence and related patterns showing only a small number of nucleotide changes, while the 16-NE categories included Asian-American (AA), Native-American (NA-1), and African (AF-1 and AF-2).

In the secondary analysis restricted to 16-E SCC cases, the variants were subdivided into 16-EP (prototype), 16-EV (European prototype sequence with a small number of additional nucleotide differences), and 16-EV-G (European sequence with the common 350T>G substitution; cases with nucleotide changes in addition to 350T>G were also included).

### Statistics

Tabular analyses were performed using chi square and Fisher's exact and Cochran-Mantel-Haenszel trend tests. Statistical significance was assigned to 2-sided probability values < .05.

## Competing interests

The authors declare that they have no competing interests.

## Authors' contributions

REZ: overall leader for this effort including data generation, data analysis and writing of the manuscript; ET: performed chart reviews and patient follow-up; NW: current team leader for SUCCEED who contributed a subset of patients, participated in data interpretation and in writing of manuscript; CM: performed chart reviews and patient follow-up; RAA: technical supervisor responsible for laboratory analyses; RS: developed HPV16 variant methodology for this study; STD: oversaw genotyping and HPV16 variant methodology; MAG: contributed patients including SUCCEED patients for this study; SSW: original team leader for SUCCEED at NCI, contributed cases for this study; JW: contributed patients including SUCCEED cases; MS: original co-team leader for SUCCEED, contributed cases to this study, provided statistical analysis and data interpretation. All co-authors participated in the preparation of this manuscript and approved the final version.
